# Fetal liver hematopoiesis: from development to delivery

**DOI:** 10.1186/s13287-021-02189-w

**Published:** 2021-02-17

**Authors:** Kyle Lewis, Momoko Yoshimoto, Takanori Takebe

**Affiliations:** 1 Center for Stem Cell & Organoid Medicine (CuSTOM), Cincinnati Children’s Hospital Medical Center, Cincinnati, OH 45229 USA; 2 Division of Gastroenterology, Hepatology and Nutrition and Developmental Biology, Cincinnati Children’s Hospital Medical Center, Cincinnati, OH 45229 USA; 3 Department of Pediatrics, University of Cincinnati College of Medicine, Cincinnati, OH USA; 4 Institute of Molecular Medicine, McGovern Medical School, University of Texas Health Science Center at Houston, Houston, Texas 77030 USA; 5 Institute of Research, Tokyo Medical and Dental University 1-5-45 Yushima, Bunkyo-ku, Tokyo, 113-8510 Japan; 6 Communication Design Center, Advanced Medical Research Center, Yokohama City University, Kanazawa-ku 3-9, Yokohama, Kanagawa 236-0004 Japan

**Keywords:** Hematopoietic stem cells, Fetal liver, Niche, Induced pluripotent stem cell (iPSC), Fetal hematopoiesis, Differentiation

## Abstract

Clinical transplants of hematopoietic stem cells (HSC) can provide a lifesaving therapy for many hematological diseases; however, therapeutic applications are hampered by donor availability. In vivo*,* HSC exist in a specified microenvironment called the niche. While most studies of the niche focus on those residing in the bone marrow (BM), a better understanding of the fetal liver niche during development is vital to design human pluripotent stem cell (PSC) culture and may provide valuable insights with regard to expanding HSCs ex vivo for transplantation. This review will discuss the importance of the fetal liver niche in HSC expansion, a feat that occurs during development and has great clinical potential. We will also discuss emerging approaches to generate expandable HSC in cell culture that attain more complexity in the form of cells or organoid models in combination with engineering and systems biology approaches. Overall, delivering HSC by charting developmental principles will help in the understanding of the molecular and biological interactions between HSCs and fetal liver cells for their controlled maturation and expansion.

## Introduction

Hematopoietic stem cells (HSCs) are a unique cell population that, in addition to self-renewal, is capable of giving rise to all the types of cells in the blood system. Clinically, this has translated into applications such as bone marrow (BM) and umbilical cord blood (UCB) transplants for patients with various hematological diseases [1, 2]. However, in order to meet the cell number required for adult patients, establishing a method of ex vivo expansion of HSCs, particularly from UCB, has been extensively explored, but still remains a challenge. HSCs usually reside in the special microenvironment of the bone marrow referred to as a niche, which supports the HSC self-renewal ability, and once HSCs leave the niche they easily lose the ability for self-renewal and either start differentiation upon cytokine signaling or undergo apoptosis. As such, decades of laboratory studies in trying to recapitulate the niche in-a-dish that permits HSCs self-renewal in vitro has proven exceedingly difficult with limited success [3].

Induced pluripotent stem cell (iPSC)-derived HSCs would offer another alternative source for transplantation. To derive HSCs from iPSCs in vitro without transgene over-expression, it is essential to understand the developmental mechanisms through which the first HSCs are produced and expand during fetal organogenesis since iPSC differentiation recapitulates aspects of embryogenesis. Importantly, the biological and molecular characteristics of fetal HSCs are considerably different from the adult HSCs. In particular, fetal HSCs expand tremendously without developing leukemia due to unique resilience mechanisms with, for example, an increased expression of DNA repair and antioxidant genes [4]. Thus, producing fetal-like HSCs and providing a microenvironment that enables fetal HSCs to expand is a desirable approach for the clinical application to transplantation therapy.

During fetal development, hematopoiesis occurs in multiple waves throughout the developing embryo and fetus, including extraembryonic yolk sac (YS), the para-aortic region of the embryo, fetal liver, and placenta before eventually homing to the bone marrow where it occurs just before birth. The first hematopoiesis is observed in the YS as early as embryonic day (E) 8.0 in mouse and Carnegie Stage (CS) 8 in humans, often referred to as primitive hematopoiesis, producing embryonic type erythroblasts that have large nuclei and express embryonic globin genes, and primitive type macrophages. The following wave is consisting of YS and embryo-derived adult type hematopoiesis that produces erythro-myeloid progenitors (EMPs) and lymphoid precursors. Finally, the first BM-repopulating HSCs are produced in the aorta-gonad-mesonephros (AGM) region at E10–11/CS13–16. These de novo HSCs seed the fetal liver and the placenta around E11–12/CS16–17 and expand to support hematopoiesis after birth [5, 6]. In the meantime, YS-derived EMPs are considered to seed the liver as well to support hematopoietic homeostasis during embryo development. The fetal liver is the major hematopoietic organ during development, supporting active erythro-myeloid hematopoiesis and HSC expansion. In this sense, the fetal liver niche marks a unique site for understanding the cell cycle dynamics of developing HSCs in contrast to the quiescent state of adult bone marrow HSCs [7].

While most studies of the HSC niche involve those residing in the BM, a better understanding of the fetal liver niche is essential to recapitulate it in vitro and may provide clinically significant information with regard to expanding HSCs ex vivo for transplantation. Organoid research, particularly liver organoids, is a rapidly growing field, in which cells are directly taken from patients or differentiated from iPSCs (reviewed elsewhere [8]). These organoids consist of multiple cell types and develop in a spatiotemporal manner that more closely resembles in vivo organs compared to simple 2D cultures. Of particular interest with regard to hematopoiesis may be the potential for future liver organoid systems to provide a niche for HSC development and expansion in vitro*.* This review will explore the stages of hematopoiesis around the time of fetal liver development while focusing on work done in mice except where noted, as well as discuss the current state of research into culturing HSCs and attempts at ex vivo expansion.

## HSC emergence and seeding the fetal liver

The first HSC emergence specifically takes place in the endothelial cells of the dorsal aorta as hemogenic endothelial cells (HECs) lining the vessel undergo endothelial to hematopoietic transition (EHT) [9, 10]. During this process, HSCs or precursors of HSCs (referred to as pre-HSCs) bud off from the surrounding endothelium where they enter the vascular network [11]. This process has been shown to be dependent on Runx1 expression, an indispensable transcriptional factor for blood cell production from HECs [12]. Notch signaling is also indispensable for HSC generation from HECs and regulates fate determination of ECs into arterial ECs or hematopoietic cells [13]. Notch signaling in turn results in several downstream transcription factors upregulated in the developing HSC including RUNX1 and GATA2 that are essential during EHT [14, 15]. After developing from the AGM, HSCs eventually home to the fetal liver where they settle in a niche microenvironment. The complete mechanism of how this settlement occurs is still not fully understood. It has been reported that β1 integrin is essential for HSC colonization to the fetal liver shown by using β1^−/−^ chimera mouse models where β1^−/−^ HSCs failed to seed the fetal liver while the production of HSCs was not impaired [16, 17]. It is of interest that E14.5 HSCs migrate in response to a gradient of CXCL12, a key chemokine attracting adult HSCs to BM, in vitro, and that the combination of stem cell factor (SCF) and CXCL12 showed enhanced migratory response of FL HSCs, while adult BM HSCs responded to only CXCL12 [18]. These results suggest that SCF and CXCL12 play a role in retaining HSCs in the fetal liver. A full understanding of these specific signaling pathways and cellular interactions at the earliest emergence of definitive hematopoiesis is crucial to recreating them through in vitro models.

## The fetal liver environment as an HSC niche

**Fig. 1:**
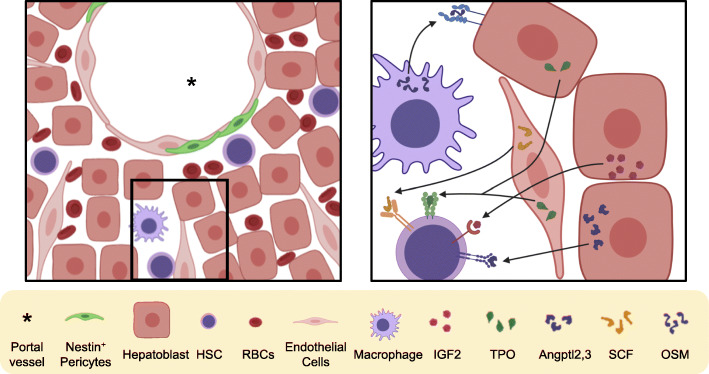
Schematic of HSC niche in the fetal liver describing cell types and molecular cross talk. HSC receive IGF2, TPO, and angptl2 and 3 from hepatocytes and SCF from endothelial cells. At the same time, macrophages produce OSM to promote maturation of hepatocytes

The developing liver is made of a mixture of cell types including both hepatic and hematopoietic lineages (Fig. 1). The liver originally arises from the liver bud after foregut patterning of the definitive endoderm (reviewed elsewhere [19]). The hematopoietic system, however, arises from the mesoderm, and thus, HSCs and other hematopoietic cells are not produced de novo in the fetal liver, but colonize the fetal liver [20]. Upon colonizing the fetal liver, HSCs undergo massive expansion up to 38-fold from days E12 to E16 in mice which corresponds to the second trimester in humans [5]. This is in stark contrast to their relative quiescence during adulthood. The fetal liver provides HSCs with a unique microenvironment to support such expansion. One of the most important aspects of the fetal liver niche for HSCs is cell-cell interactions, either directly, or through cytokine signaling.

Understanding the specific location and cell types that form the HSC niche in the fetal liver has long been an area of research. Early hematopoiesis in the fetal liver occurs prior to the full maturation of the hepatic vascular system. Because of this, hematopoietic cells including HSCs are found in the liver parenchyma where they are able to directly interact with various cell types (Fig. 1). It has been shown that HSCs in the fetal liver are found in close association with Nestin^+^ stromal cells adjacent to portal vessels [21]. However, it is not clear whether Nestin^+^ cells secrete cytokines or have direct cross talk with HSCs.

In order to determine the factors and stromal cells that support HSC expansion, fetal liver stromal cells were isolated and tested their HSC supportive ability [22, 23]. AFT024, a single stromal cell clone from fetal liver, maintained BM and fetal liver HSCs for as long as 5–7 weeks, providing a niche for expansion of HSC [24]. However, the mechanisms for fetal liver HSC maintenance in vitro have yet to be elucidated due to the changing characteristics of the stromal cells over time.

From freshly isolated fetal liver stromal cells, angiopoietin-like 2 and 3 (angptl2, 3) have been identified to expand HSCs [23]. The following study by the same group has demonstrated that SCF^+^Dlk1^+^ fetal liver stromal cells express angptl3, in addition to alpha-fetoprotein (AFP, a marker of fetal hepatoblasts), CXCL12, and essential hematopoietic cytokines including SCF, thrombopoietin (TPO), and insulin-like growth factor 2 (IGF2). SCF^+^Dlk1^+^ cells maintained E15 fetal liver HSC repopulating ability during 4 days of co-culture. Others have reported that Dlk1^+^ fetal hepatic progenitors secrete SCF and Epo, confirmed by ELISA assays and immunostaining [25]. Thus, it is likely that Dlk1^+^ fetal hepatoblasts secrete essential hematopoietic cytokines and support HSC expansion. Since Tie-2 and Angiopoietin-1 signaling has a critical role in maintaining HSC quiescence in the BM niche, it will be interesting to examine the interaction of fetal liver HSCs with angptl3 during expansion in the fetal liver niche [26].

Another main type of stromal cells in the liver is endothelial cells. As previously mentioned, HSCs are found in close proximity to endothelial cells in the developing fetal liver. More recently, it has been observed that endothelial cell-selective adhesion molecule (ESAM) expressed on endothelial cells and HSCs plays an essential role in this interaction in the fetal liver and is crucial to erythropoiesis [27]. Endothelial cells in the fetal liver have been reported to express membrane-bound SCF and support erythropoiesis, but plays a role in HSC expansion as well [28]. Traditionally, stem cell factor mutant mice (Sl/Sl mice) are known to be embryonic lethal due to fetal anemia [29] and SCF-signaling has been shown to be indispensable for definitive hematopoiesis [30]. In the Sl/Sl homozygous fetal liver, the number of HSCs is dramatically reduced, but still maintains a small increase during E13–15, suggesting the presence of HSC self-renewal but with impaired expansion in the absence of SCF [29]. These results indicate the critical roles of SCF in the proliferation of FL HSCs.

Each of the hepatic and hematopoietic lineages develops together during the process of fetal liver hematopoiesis. This co-differentiation system allows for a complex interaction network that is still not fully described. However, it is known that the niche environment promotes differentiation or maturation to both lineages through cellular cross talk. During fetal hematopoietic development, macrophages release Oncostatin M (OSM) which is important for hepatocyte differentiation [31]. Similarly, megakaryocyte progenitors have been reported to promote hepatoepithelial cell development into hepatocytes in E11.5 fetal liver through cell to cell contact [32]. Conversely, the effects of hepatic stromal cells on fetal liver hematopoiesis have also been reported. Gli3 is a protein in the sonic hedgehog (SHH) pathway that often acts as a transcriptional repressor and is expressed in hepatic stromal cells. It has been reported that B cell development in Gli3^−/−^ mouse fetal liver was reduced at multiple stages due to upregulated SHH in non-hematopoietic cells [33]. This report suggests that Gli3 activity in the fetal liver stroma promotes normal B cell development by repressing Shh.

Bile acids have also been shown to play a key role in HSC expansion in the fetal liver [34]. Despite increased protein production during expansion, HSC in the fetal liver are resilient against unfolded/misfolded protein accumulation. CYP27A1 is an essential enzyme for the metabolism of cholesterol into bile acids and, in doing so, plays a key role in bile acid synthesis. Careful analysis of pregnant heterozygous and homozygous CYP27A1 mutant mice revealed a unique role for the maternal and embryonic bile acids in protecting HSC expansion in the fetal liver. Interestingly, treatment with bile acids in vitro maintained engraftment capability of cultured FL HSCs, suggesting a potential use for bile acids in ex vivo expansion of HSC.

The interaction between hepatoblasts and HSCs is also seen in human fetal liver. Human fetal liver CD34^lo^CD133^lo^ stromal cells have been reported to support CD34^hi^CD133^hi^ fetal liver HSCs [35]. CD34^lo^CD133^lo^ cells were isolated from human fetal liver (15–23-week gestation), shown to express AFP, CK18, CK19, and EpCAM (CD326), like fetal hepatocyte progenitors, and differentiated into hepatocytes both in vitro and vivo, expressing albumin, HNF1A, and HNF1B [36]. CD34^lo^CD133^lo^ cells maintained SCID-repopulating ability of CD34^hi^CD133^hi^ fetal liver HSCs after 7 days co-culture [35]. These results suggest that CD34^lo^CD133^lo^ cells are immature hepatocyte progenitors and support fetal liver hematopoiesis. In another report, human fetal liver cells have also been cultured for short term (6 days) and were able to maintain hepatic, endothelial, and hematopoietic lineages [37]. These cells were then able to be engrafted into immune-deficient mice with successful all three lineages.

Thus, it is likely that the crosstalk between hepatoblasts and hematopoietic cells leads both hepatoblast maturation into hepatocytes as well as HSC expansion. Insights gained from these co-culture experiments may help design expansion systems without the need for fetal cells. Future experiments may focus on iPSC-derived hepatic/HSC cultures, which often remain fetal in nature and may be a useful source for modeling HSC in the fetal liver microenvironment.

## Directed differentiation to HSC and in vitro expansion

One of the more obvious and long attempted methods for in vitro production of HSC for transplant is directed differentiation from embryonic stem (ES) cell or iPSC sources (reviewed elsewhere [38]). In addition to this, mature cells of other lineages such as endothelial or committed hematopoietic cells have been reprogrammed to HSC [38]. In these experiments, careful analysis of which genes are expressed during critical times of HSC development has allowed for targeted approaches that contain only a small number of genes to be overexpressed allowing for directed differentiation. Recent success has been seen in directed differentiation of iPSC to HSC using a combination of morphogen-mediated differentiation alongside overexpression of seven transcription factors [39]. Additionally, a set of four transcription factors has been used for direct conversion of adult endothelial cells into functional HSC [40]. Unfortunately, transgene overexpression in these cells largely precludes them from clinical use. Additionally, it is still unclear whether HSC created through these methods fully mature into adult-like HSC or instead maintain a fetal gene signature and phenotype.

Another important factor is the selection of potential media for use in fetal liver HSC cell culture experiments. There are several commercially available media that focus on expansion culture of HSC, regardless of cell source; however, these provide limited expansion and are not yet in use for clinical expansion of HSC. Furthermore, culture of fetal liver HSC may have different media requirements, especially if liver cells are included in a co-culture system. Differentiation of hepatic cells from iPSC or embryonic stem cells (ESC)  requires a series of growth factors including BMP4, FGF2, HGF, and Oncostatin M [41–43]. Table 1 describes potential cytokines and growth factors that would be required for use in maintaining both lineages. In the co-culture system, the selection and dosage of soluble factors that promote either HSC expansion or hepatocyte maintenance would affect the other lineage's behavior, positively or negatively. Therefore, careful optimization will be required based on the purpose.

**Table 1:** Description of cytokines and growth factors produced in vivo that may be required for fetal liver HSC culture in the presence of hepatic cells. *STM* septum transversum mesenchyme, *HSPC* hematopoietic stem and progenitor cells

Cytokine/growth factor	Cell type promoted	Cell of origin in vivo	In vitro concentration ranges	
SCF	HSPC	Endothelial cells	1–100 ng/ml	[22, 23, 25, 29, 48]
TPO	HSPC, Megakaryocytes	Endothelial/hepatocytes	1–100 ng/ml	[23, 48]
IGF2	HSPC	Hepatocytes	20 ng/ml	[22, 23, 55, 56]
ANGPTL2,3	HSPC	Hepatocytes	100 ng/ml	[22, 23]
BMP4	Hepatocyte	Cardiac mesoderm/STM	20 ng/ml	[41, 57]
FGF2	Hepatocyte	Cardiac mesoderm/STM	10 ng/ml	[41, 57]
HGF	Hepatocyte	Mesenchyme	10–20 ng/ml	[41, 42]
Oncostatin M	Hepatocyte	Macrophages, Stellate cells	10–20 ng/ml	[41, 43]

## Fetal and adult HSC: differences in biology and gene signature

While fetal liver HSCs home to the bone marrow around birth and settle in the BM niche, there are differences in biological and molecular characteristics between fetal liver and adult BM HSCs, including cell surface markers, lineage differentiation bias upon transplantation, gene expression patterns, and cell cycle rates [44, 45]. HSC in the fetal liver undergo a period of massive expansion marked by continual cell cycling while adult HSCs remain in a state of quiescence and are only activated to maintain homeostatic levels or in the case of infection or injury. Because of this, these two types of cells have different levels of oxygen consumption and metabolic rates [4]. This leads to an increase in oxidative phosphorylation and reactive oxygen species production in FL HSCs. Interestingly, FL HSCs exhibit a unique resilience in such conditions due to an increased expression of DNA repair and antioxidant genes [4].

The gene signatures of HSC play a key role in their trafficking during development as well. The exact gene signature of HSC at various stages of development is still an area of current research, and recent technological advances in single cell transcriptomic analyses are revealing a huge amount of data to unravel this mystery. Recently, a combination of single cell RNA sequencing (scRNAseq) and ATACseq was used in fetal HSCs from AGM, fetal liver, and bone marrow to analyze gene signature differences including epigenetic changes in mice and humans. Single cell sequencing of the human AGM region has revealed two separate populations of early HSCs, similar to the separate waves of early hematopoiesis in model organisms, with the earlier one lacking arterial markers [46]. Sequencing in the fetal liver has shown a large number of genes that are differentially regulated compared to their adult bone marrow counterparts, many of which are associated with altered cell cycle [47]. Furthermore, analysis of the genome revealed that adult HSCs have an increase in chromosomal compartment boundaries and different interactions between promoter and transcription factors compared to fetal HSCs [44]. Such detailed analyses allow us to better characterize fetal liver HSCs and may lead to better ways to model the niche environment necessary to induce ex vivo proliferation as well as provide essential controls for comparison of engineered HSCs.

## Engineered niches mimicking adult BM and fetal liver

Many attempts at expanding HSC ex vivo utilize the cytokines present in the fetal liver, with SCF and TPO being the main two. Recently, a breakthrough in ex vivo HSC culture was reported with murine HSCs capable of being expanded between 236 and 899-fold [48]. This study used media supplemented with high levels of TPO and low levels of SCF in addition poly vinyl alcohol as a novel replacement for serum albumin. However, this has not yet been reproduced in human HSC.

**Fig. 2:**
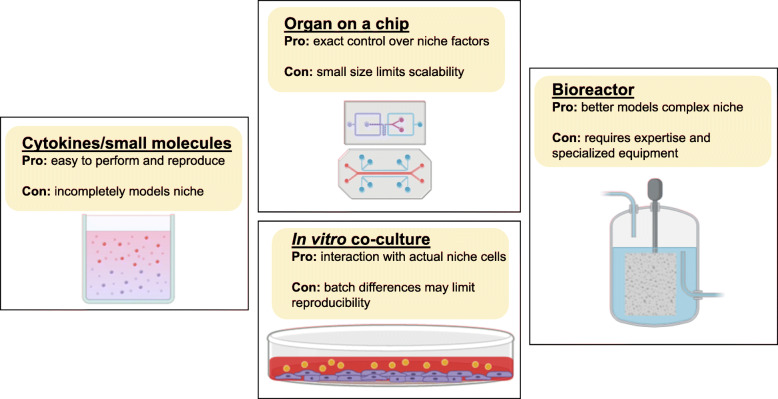
Diagram of different methods currently used for ex vivo HSC expansion

In the field of bioengineering, a combination of engineering approaches has been utilized to create a human HSC niche for ex vivo culture (Fig. 2). However, most attempts have focused on modeling the adult bone marrow niche. In one such attempt, a bone marrow-on-a-chip method was implemented to mimic the adult HSC niche [49]. While HSC were able to be maintained, due to its limited size, such systems are better suited for drug screening rather than HSC expansion. In another particularly promising attempt at ex vivo niche recreation, a bone marrow niche environment was engineered using a perfused bioreactor system [50]. This system utilized a combination of engineered extracellular matrix (ECM) and perfused media to model the adult bone marrow niche. With it, researchers were able to see an increase in numbers of HSPC after culture. These culture systems could be adapted to contain fetal-like hepatic cells from iPS cultures to better model the fetal liver HSC niche.

It has been shown that the HSC in the fetal liver exist in close proximity to certain cells, specifically around the portal vessels [21]. Future in vitro models may need to exhibit tight control of the location of HSCs relative to other cells in the culture. Recent advances in biopolymers and hydrogels have allowed the incorporation of ECM into cell culture systems containing cells and immobilized soluble factors to maintain a higher level of control over cells without the complexity of a bioreactor. One such system recently published used a mesh system to immobilize HSC and mesenchymal stromal cells in close enough contact to induce cellular crosstalk [51]. Such a system could also be utilized in conjunction with hepatic cells to provide an in vitro fetal liver microenvironment.

During embryogenesis, hemodynamic flow (shear stress) is critical for proper development of hematopoietic cells in vivo [52]. Microfluidics systems are uniquely able to emulate this aspect. Recently, a microfluidics-based ex vivo “fetal liver-on a-chip” model has been developed to observe homing behavior of HSCs and interaction between HSCs and fetal liver cells using two-photon microscopy [53]. This system will help in the understanding of the molecular and biological interactions between HSCs and fetal liver cells for their maturation and expansion.

Another possibility for niche creation is through differentiation of iPSC or ESC  into a complex multilineage hepatic culture. Recently, iPSC were engineered to overexpress GATA6 to induce differentiation into the three germ layers [54]. These iPSC cultures were guided to definitive endoderm and subsequently hepatic differentiation. Interestingly, the resulting hepatic cultures expressed markers for fetal liver cells and also produced hematopoietic cells, likely through EHT of residual mesodermal populations. Such systems provide a potential model for discovering new interactions and pathways between hepatic and hematopoietic cells.

## Conclusion

This review has discussed the importance of the fetal liver niche in HSC expansion, a feat that occurs during development and has great clinical potential, yet has thus far proven difficult to recapitulate for ex vivo HSC expansion. Recent studies have increasingly honed in on specific cytokines cocktails for culturing HSC, and while some experiments are looking at modeling the HSC niche in culture with co-culture or ECM systems, many of these focus on the BM niche. This might not be ideal for the large-scale expansion that is desired and present in the fetal liver because iPSC-derived HSCs, if produced, would have similar characteristics of fetal HSCs. Current in vitro co-culture systems involving fetal liver HSCs largely involve with primary fetal cells which limits experiments to mouse cells [22, 37]. As new innovative approaches are developed in cell culture, future systems may obviate the need for primary tissue in the form of iPS-derived hepatic co-culture or organoid models in combination with the previously mentioned systems to induce expansion of HSC.

## Data Availability

Please contact author for data requests.

## Abbreviations

HSC: Hematopoietic stem cells
iPSC: Induced pluripotent stem cells
ECM: Extracellular matrix
ESCs: Embryonic stem cells
BM: Bone marrow
HECs: Hemogenic endothelial cells
EHT: Endothelial to hematopoietic transition
YS: Yolk sac
